# Dcm methylation is detrimental to plasmid transformation in *Clostridium thermocellum*

**DOI:** 10.1186/1754-6834-5-30

**Published:** 2012-05-06

**Authors:** Adam M Guss, Daniel G Olson, Nicky C Caiazza, Lee R Lynd

**Affiliations:** 1 Thayer School of Engineering, Dartmouth College, 8000 Cummings Hall, Hanover, NH 03755, USA; 2 Biosciences Division, Oak Ridge National Laboratory, Oak Ridge, TN 37831, USA; 3 Mascoma Corporation, 67 Etna Rd, Suite 300, Lebanon, NH 03755, USA; 4 Current address: Synthetic Genomics Inc, La Jolla, CA 92037, USA

**Keywords:** *Clostridium thermocellum*, DNA methylation, Transformation efficiency, Consolidated bioprocessing

## Abstract

**Background:**

Industrial production of biofuels and other products by cellulolytic microorganisms is of interest but hindered by the nascent state of genetic tools. Although a genetic system for *Clostridium thermocellum* DSM1313 has recently been developed, available methods achieve relatively low efficiency and similar plasmids can transform *C. thermocellum* at dramatically different efficiencies.

**Results:**

We report an increase in transformation efficiency of *C. thermocellum* for a variety of plasmids by using DNA that has been methylated by *Escherichia coli* Dam but not Dcm methylases. When isolated from a *dam*+*dcm*+*E. coli* strain, pAMG206 transforms *C. thermocellum* 100-fold better than the similar plasmid pAMG205, which contains an additional Dcm methylation site in the *pyrF* gene. Upon removal of Dcm methylation, transformation with pAMG206 showed a four- to seven-fold increase in efficiency; however, transformation efficiency of pAMG205 increased 500-fold. Removal of the Dcm methylation site from the pAMG205 *pyrF* gene via silent mutation resulted in increased transformation efficiencies equivalent to that of pAMG206. Upon proper methylation, transformation efficiency of plasmids bearing the pMK3 and pB6A origins of replication increased ca. three orders of magnitude.

**Conclusions:**

*E. coli* Dcm methylation decreases transformation efficiency in *C. thermocellum* DSM1313. The use of properly methylated plasmid DNA should facilitate genetic manipulation of this industrially relevant bacterium.

## Introduction

The transition to a sustainable resource base is one of the largest challenges facing humanity [1], with transportation being a among the largest and fastest-growing energy demands [2]. While cellulosic biomass is a promising feedstock for the generation of renewable transport fuels, the cost of enzymatic hydrolysis of cellulose to soluble sugars is currently too high to be economically viable [3]. Combining the steps of enzyme production and sugar fermentation in a one-step process called consolidated bioprocessing (CBP) has the potential to address this limitation but requires the development of an organism that both degrades cellulose efficiently and produces fuel at high yield and titer [4].

*Clostridium thermocellum* is a thermophilic, anaerobic member of the Firmicute phylum of bacteria that specializes in cellulose degradation. *C. thermocellum* serves as a model organism for the study of microbial cellulose hydrolysis because of its cellulosome, an extracellular enzyme complex that tethers the cell to crystalline cellulose and mediates its rapid solubilization. Furthermore, *C. thermocellum* produces ethanol as one of its fermentation products and thus has potential for consolidated bioprocessing.

The nascent state of genetic tools has hindered both fundamental and applied studies of *C. thermocellum*. However, recent advances have started to remedy this situation. Introduction of heterologous DNA by electrotransformation has been demonstrated using a custom electroporator with custom cuvettes [5] as well as with standard electroporation equipment [6]. Further, positive and negative selection systems have been developed and used to demonstrate gene replacement [7].

Although genetic manipulation of *C. thermocellum* is now possible, we have observed that transformation efficiency can vary greatly between plasmids, even when they are very similar. Due to the difficulties still involved in genetic modification of *C. thermocellum*, we aimed to understand the cause of this plasmid-to-plasmid variation in transformation efficiency. Many barriers to transformation have been discovered in other organisms, one principle being improper DNA methylation of the plasmids to be transformed. Indeed, *E. coli* methylation of plasmid DNA has been shown to inhibit transformation in a variety of organisms [8-10]. However, *C. thermocellum* ATCC 27405 has an *Mbo*I-type restriction system that is blocked by *E. coli* Dam methylation [11], suggesting that at least some *E. coli* DNA methylation may be required for transformation in *C. thermocellum*. We therefore examined the effect of altering the *E. coli* methylation of plasmid DNA on *C. thermocellum* transformation efficiency.

## Results

**Figure 1:**
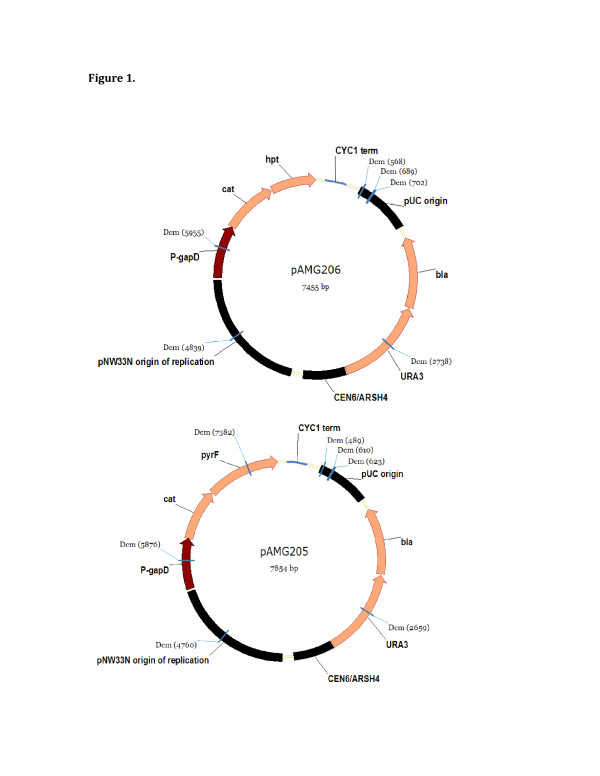
**Plasmid maps of pAMG205 and pAMG206.** The only difference between these plasmids is the presence of either the *C. thermocellum pyrF* gene or the *T. saccharolyticum hpt* gene. Dcm methylation sites are highlighted. CYC1 term, *Saccharomyces cerevisiae* CYC1 transcriptional terminator; pUC origin, origin of replication from pUC19; bla, b-lactamase; URA3, *Saccharomyces cerevisiae* URA3 gene; CEN6/ARSH4, low copy origin of replication in *Saccharomyces cerevisiae*; pNW33N ori, origin of replication from pNW33N; repB, encodes putative protein responsible for plasmid replication initiation; putative mob/pre fragment, encodes putative protein with homology to the Mob/Pre family involved in plasmid mobilization and recombination; P-gapD, promoter region of the *C. thermocellum gapD* gene; cat, chloramphenicol acetyltransferase; *pyrF*, *C. thermocellum* orotidine ′-phosphate decarboxylase gene; *hpt*, *T. saccharolyticum* hypoxanthine phosphoribosyltransferase gene.

The plasmids pAMG205 and pAMG206 (Figure 1) differ only by a single gene, the *C. thermocellum pyrF* gene or the *T. saccharolyticum hpt* gene, respectively. Despite high similarity, plasmid pAMG205 transforms wild type *C. thermocellum* (i.e., wild type at the *pyrF* and *hpt* loci) at very low efficiency when isolated from standard cloning strain *E. coli* Top10, whereas plasmid pAMG206 transforms at ca. 100-fold higher efficiency under identical conditions (Table 1). We hypothesized that DNA methylation might account for this difference in transformation efficiency. Therefore, the plasmids were each isolated from an *E. coli* strain that lacks both the Dam and Dcm DNA methylases. In this case, both plasmids transform *C. thermocellum* very poorly (ca. 500-fold lower efficiency for pAMG206 compared to *dam*+*dcm*+DNA; Table 1). This result indicates that at least one of the E. coli DNA methylases is important for DNA transformation into *C. thermocellum*.

**Table 1:** **Number of transformants isolated when *E. coli *methylation is varied**

		**Average number of transformants per µg DNA (range)**	
***E. coli *host**^**a**^	**Methylation**^**b**^	**pAMG205**	**pAMG206**
Top10	Dam+, Dcm+	4 (0−14)	570 (95−630)
C2925	Dam-, Dcm-	1 (0−4)	1 (0−2)
C2925+i.v. Dam^c^	Dam+, Dcm-	2200 (960−4800)	1900 (820−4200)
BL21	Dam+, Dcm-	2600 (680−5900)	4400 (1100−9500)

^a^*E. coli* strain from which plasmid DNA was isolated.

^b^State of plasmid methylation by *E. coli* Dam and Dcm DNA methylases.

^c^Plasmid DNA was methylated in vitro by *E. coli* Dam methylase.

To determine whether Dam methylated DNA improves transformation efficiency, unmethylated plasmid DNA was Dam methylated in vitro and transformed into *C. thermocellum*. Dam methylated pAMG206 transformed *C. thermocellum* ca. 1000-fold more efficiently than unmethylated DNA and 4-fold more efficiently than DNA isolated from Top10 (Table 1). Interestingly, pAMG205 that had been methylated in vitro transformed *C. thermocellum* just as well as pAMG206 and 500-fold better than pAMG205 isolated from Top10. To further test the idea that Dcm methylation might reduce the efficiency of transformation in *C. thermocellum*, pAMG205 and pAMG206 were isolated from *E. coli* BL21 (DE), which is *dam*+*dcm*-. Plasmids isolated from BL21 transformed *C. thermocellum* at comparable efficiencies both to each other and to that observed when the DNA was Dam methylated in vitro (Table 1). These findings suggest not only that Dam methylation is beneficial but also that Dcm methylation is detrimental to *C. thermocellum* transformation efficiency.

Upon examination of the DNA sequence of pAMG206, six Dcm methylation sites (CCWGG, where W=A or T) were identified, all of which were in the 6.8 kilobase region common to both plasmids, but pAMG205 has an additional Dcm methylation site located in the *pyrF* gene (Figure 1). Based on the above results, we hypothesized that this site is responsible for the difference in transformation efficiency between the two plasmids. To test this hypothesis, a silent mutation was introduced into the *pyrF* gene of pAMG205 that eliminated the Dcm methylation site (*pyrF* C555G, resulting in ACC→ACG, maintaining T185 but converting Dcm site CCAGG to non-Dcm site CGAGG), leaving the same six sites that are present in pAMG206. The resulting plasmid, pAMG205△dcm7, transforms *C. thermocellum* at the same efficiency as pAMG206 (Table 2), indicating that this Dcm methylation site is responsible for the difference in transformation efficiency between pAMG205 and pAMG206.

**Table 2:** **Number of transformants isolated when Dcm methylation site in *pyrF *is removed by silent mutation**

		**Average number of transformants per µg DNA (range)**		
***E. coli *host**^**a**^	**Methylation**^**b**^	**pAMG205**	**pAMG206**	**pAMG205Δdcm#7**
Top10	Dam+, dcm+	2 (0−6)	160 (95−240)	220 (60−520)
BL21	Dam+, dcm-	1200 (680−1700)	1400 (1100−1900)	1500 (840−2200)

^a^*E. coli* strain from which plasmid DNA was isolated.

^b^State of plasmid methylation by *E. coli* Dam and Dcm DNA methylases.

To determine if the negative effect of Dcm methylation is a general phenomenon or specific to pNW33N-based plasmids such as pAMG205 and pAMG206, plasmids with different origins of replication were tested for Dcm-dependent decrease in transformation efficiency (Table 3). Plasmids containing the pMK3 [12] and pB6A [13] replicons were able to transform *C. thermocellum* well when isolated from the *dcm*- *E. coli* BL21, but poorly or not at all not when isolated from the *dam*+*dcm*+*E. coli* Top10 (Table 3).

**Table 3:** Number of transformants isolated when varying Dcm methylation of plasmids with different origins of replication

			**Average number of transformants per µg DNA (range)**	
**Plasmid**^**a**^	**Replication origin**	**# Dcm sites**^**b**^	**Top10 DNA**^**c**^	**BL21 DNA**^**c**^
pMU1117	pMK3	11	1 (0−3)	660 (220−1300)
pMU1054	pB6A	9	0 (0)	2600 (780−5600)

^a^Plasmid sizes: pMU1117, 9018 bp; pMU1054, 6833 bp.

^b^Number of *E. coli* Dcm methylation sites (CCWGG) present in the plasmid.

^c^*E. coli* strain from which plasmid DNA was isolated.

## Discussion

Here we demonstrate that Dam methylation increases but Dcm methylation decreases transformation efficiency in *C. thermocellum* DSM 1313. Therefore, isolating DNA from a *dam*+*dcm*- *E. coli* strain such as BL21 allows for higher transformation efficiency overall while also eliminating the plasmid-to-plasmid variation observed when DNA was isolated from *E. coli* Top10. This modification in transformation protocol should accelerate genetic analysis and engineering in this organism for enhanced cellulosic biofuel production.

The presence of *E. coli* Dam methylation was important for transformation of *C. thermocellum* DSM1313, suggesting that it has a functional homolog of the MboI-type restriction system present in *C. thermocellum* ATCC27405, likely encoded by *Clo1313_2274*. It is unclear why pAMG206, with six Dcm methylation sites, transforms *C. thermocellum* relatively well when isolated from a *dam*+*dcm*+*E. coli* strain, while the additional Dcm methylation site in the *pyrF* gene reduces the transformation efficiency by nearly three orders of magnitude. One explanation is that *C. thermocellum* DSM 1313 could encode a restriction system that targets methylated DNA with a recognition sequence that only overlaps some Dcm methylation sites. While no palindromic sequence was identified at the *pyrF* Dcm-methylation site, some restriction enzymes recognize non-palindromic sequences [14] and therefore the recognition site could still overlap with the Dcm methylation site. Furthermore, while Type II restriction systems are rarely specific for methylated DNA, Type IV restriction systems are common and target methylated DNA for cleavage. Indeed, the recently published genome of *C. thermocellum* DSM1313 revealed the presence of a Type IV restriction system (Clo1313_2373, [15,16]). Strain DSM1313 also encodes a putative Type III restriction system, which typically requires multiple, non-palindromic sites and rarely gives complete digestion in vitro. If Dcm methylation were to overlap and interfere with this restriction system, it could explain the drastic difference in transformation efficiency between these plasmids despite the addition of only a single additional methylation site.

While this study has demonstrated both an increase in transformation efficiency and a decrease in plasmid-to-plasmid variation in efficiency, more effort could be spent increasing transformation efficiency. For instance, further examination of DNA methylation could be fruitful. While only one restriction system has been described in *C. thermocellum* (strain ATCC 27405), New England Biolabs REBASE predicts that strain ATCC27405 encodes at least five restriction systems and strain DSM1313 encodes at least four [16]. Therefore, proper DNA methylation could further improve transformation efficiency by blocking *C. thermocellum* restriction endonucleases.

## Conclusions

In this work, the plasmid-to-plasmid variability in transformation efficiency in *C. thermocellum* was discovered to be impacted by *E. coli* Dcm methylation of the plasmid DNA. By eliminating Dcm methylation, transformation efficiency was increased by up to 1000-fold. This realization allows dramatic improvement in the usability of recently developed genetic tools, enabling both fundamental studies of microbial cellulose utilization and metabolic engineering for production of value-added products from cellulose.

## Methods

### Microbial strains and growth conditions

Yeast and bacterial strains are listed in Table 4. *Saccharomyces cerevisiae* InvSc1 was maintained on YPD medium and grown on SD-ura medium (Sunrise Science Products, San Diego, CA, USA) when selecting for the presence of URA3+ plasmids. *E. coli* strains were grown on LB medium and supplemented with chloramphenicol (12 µg/ml) or ampicillin (100 µg/ml) as required for plasmid maintenance. *C. thermocellum* DSM 1313 was grown inside a Coy anaerobic chamber (Coy Laboratory Products, Grass Lake, MI) in modified DSM122 medium [5] supplemented with 50 mM MOPS and 10 mM sodium citrate [7] at 51°C, and 10 µg/ml thiamphenicol was added when selecting for plasmid maintenance. Medium was made anaerobic via autoclaving to remove O_2_ from solution, followed by immediate transfer to the anaerobic chamber to maintain anaerobicity.

**Table 4:** Strains and plasmids

**Strain or plasmid**	**Relevant features**	**Source/reference**
Microbial strains		
*S. cerevisiae* InvSc1	uracil auxotroph	Invitrogen
*E. coli* Top10	*dam*+*dcm*+	Invitrogen
*E. coli* C2925	*dam*- *dcm*-	New England Biolabs
*E. coli* BL21 (DE3)	*dam*+*dcm*-	New England Biolabs
Plasmids		
pAMG205	oriColE1, bla, CEN6, ARSH4, URA3, PgapD-cat-pyrF, pNW33N replication origin	This study
pAMG206	oriColE1, bla, CEN6, ARSH4, URA3, PgapD-cat-hpt, pNW33N replication origin	This study
pAMG205△dcm7	pAMG205△pyrF::pyrF*	This study
pMU1054	oriColE1, bla, P-gapD-cat, pB6A origin	This study
pMU1117	oriColE1, bla, P-gapD-cat, pMK3 origin	This study

### Plasmid construction and DNA manipulation

Plasmids used in this study and their relevant features are listed in Table 4. Plasmids were constructed (Additional file 1: Table S1, Additional file 1: Table S2) using yeast gap repair cloning [17] or standard *E. coli* methods [18]. For gap repair cloning, DNA was transformed into yeast via a modified Lazy Bones protocol [19,20] and assembled into a contiguous piece of DNA via yeast homologous recombination. Plasmid DNA was isolated from yeast using Zymoprep Yeast Plasmid Miniprep II kit (Zymo Research, Orange, CA, USA) and introduced via electroporation into *E. coli* Top10 (*dam*+*dcm*+*E. coli* K12 derivative from Invitrogen, Carlsbad, CA) and via chemical competence into *E. coli* BL21 (DE3) (*dam*+*dcm*- *E. coli* B derivative; New England Biolabs, Ipswich, MA) and *E. coli* C2925 (*dam*^-^*dcm*^-^*E. coli* K12 derivative; New England Biolabs). All PCR amplified regions were sequenced at the Dartmouth Molecular Biology Core Facility to verify PCR fidelity. Plasmid DNA was purified from *E. coli* using QIAGEN Miniprep Kit. In vitro DNA methylation was carried out according to manufacturer’s instructions using *E. coli* Dam methylase (New England Biolabs). All DNA to be transformed in *C. thermocellum* was additionally purified and concentrated to 500 ng/µl using Zymo Research DNA Clean & Concentrator − 5 kit.

### *C. thermocellum* transformation

*C. thermocellum* was transformed via electroporation as described [5,6] with modifications. Briefly, 400 ml*C. thermocellum* was grown to an OD between 0.8 and 1.0, centrifuged without measures to exclude oxygen, since washing the cells in the presence of O_2_ seemed to have no impact on transformation efficiency (unpublished observations), at room temperature in a Beckman Coulter Avanti J-25 centrifuge with a JA-10 rotor at 5000 × g, and the supernatant was removed. Being careful to minimize disturbance, cell pellets were washed with 400 ml ice cold electroporation buffer prepared without measures to exclude oxygen and consisting of 250 mM sucrose, 10% glycerol, 100 µM MOPS pH 7.0, 0.5 mM MgCl_2_, 0.5 mM MgSO_4_ and centrifuged at 4000×g. The cells were rinsed and centrifuged a second time as above and brought on ice into a Coy anaerobic chamber, maintaining anaerobicity for the remainder of the transformation. Cells were resuspended in an additional 500 µl electroporation buffer and kept on ice until use. Plasmid DNA (2 µl at 500 ng/µl) was mixed with 20 µl washed cells in pre-chilled 1 mm electroporation cuvettes. The mixture was then subjected to a 1.2 kV, 1.5 msec square pulse using a BioRad GenePulser XCell. Cells were immediately resuspended in 1 ml room temperature growth medium and serial dilutions were plated with no recovery period (to ensure each colony represents a unique transformant) by mixing with 25 ml molten media+0.8% agar+thiamphenicol. Once plates had solidified, they were placed in 2.5 L AnaeroPack Rectangular Jars (bioMerieux, Durham, NC, USA) to minimize desiccation and incubated at 51°C for up to one week. Transformations were repeated at least three times, and the mean and range of efficiency is reported.

Successful transformation was confirmed by re-isolation of plasmid DNA. Briefly, chromosomal DNA was isolated from thiamphenicol resistant *C. thermocellum* transformants using the QIAGEN DNeasy kit (Qiagen, Valencia, CA) following the pretreatment protocol for DNA isolation from Gram-positive bacteria according to manufacturer’s specifications. This DNA was transformed into *E. coli* Top10 cells, re-isolated, and subjected to restriction enzyme digestion to confirm the identity of the plasmid.

## Competing interests

LL is a co-founder and active consultant for Mascoma Corporation, at which DGO and NCC were formerly employed. Mascoma has a financial interest in biomass conversion processes.

## Authors’ contributions

AMG helped conceived of the study, participated in its design and coordination, carried out the transformations, and drafted the manuscript. DGO and NCC participated in the study design and identified and constructed plasmids with different origins of replication. LRL helped conceive of the study, participated in its design and coordination, and helped to draft the manuscript. All authors read and approved the final manuscript.

## Supplementary Material

Additional file 1 **Table S1.** Construction or source of plasmids [12,13,21]. **Table S2.** Primers used in plasmid construction. Click here for file
