# Supplementary material for: Dcm methylation is detrimental to plasmid transformation in Clostridium thermocellum
Source: Biotechnol Biofuels. 2012 May 6;5:30. doi: 10.1186/1754-6834-5-30 (PMC3536630; doi:10.1186/1754-6834-5-30)
Supplement: Additional file 1 — Table S1. Construction or source of plasmids [12,13,21]. Table S2. Primers used in plasmid construction. [file 1754-6834-5-30-S1.doc]

**Supplemental Table 1.** Construction or source of plasmids

| Plasmid | Source or construction |
| --- | --- |
| pAMG202 | The 3.9 kb PmeI + SpeI fragment of pYC2/CT was combined with the PCR product of primers **Cthe_pyrF_rev+40bp_pYC2** and **Cthe_pyrF_for+40bp_cat** (amplifies *pyrF* [Cthe0951] gene from *C. thermocellum*) and the PCR product of primers **P-gapD-cat for+40bp_pYC2** and **P-gapD-cat rev+40bp_pyrF** (amplifies P-gapD-cat from pMU771) via yeast gap repair |
| pAMG203 | The 5.3 kb ZraI+SmaI fragment from pAMG202 was combined with the PCR product of primers **Tsacch-hpt-for+40bp_cat** and **Tsacch-hpt-rev+40 bp_pAMG202** (hpt gene from T. saccharolyticum) via yeast gap repair |
| pAMG205 | The 6.3 kb SpeI fragment of pAMG202 was combined with the PCR product of primers **pNW33Nori-for+40bp_pAMG202SpeI** and **pNW33Nori-rev+40bp_pAMG202SpeI** via yeast gap repair |
| pAMG206 | The 6.9 kb ZraI+SmaI fragment from pAMG205 was combined with the 1.9 kb BamHI+AscI fragment of pAMG203 via yeast gap repair |
| pAMG205Δdcm7 | The 6.9 kb ZraI+SmaI fragment from pAMG205 was combined with PCR products using pAMG202 as template with two primer sets: 1) **pyrF internal to del dcm-f** and **pYC2-CT-seq-r**, and 2) **pyrF internal to del dcm-r** and **cat-seq-out-f**. They were cloned together using yeast gap repair. |
| pB6A |  |
| pMK3 |  |
| pMU121 | pB6A was linearized with MfeI and ligated into the EcoRI site of pUC19 |
| pMU770 |  |
| pMU1054 | KpnI and XbaI fragment of pMU121 was ligated to the 1.8 kb KpnI-XbaI fragment of pMU770 containing the gapD-cat fragment |
| pMU1117 | pMK3 was linearized with BamHI and ligated to the 1.8 kb BamHI fragment of pMU770 containing PgapD-cat |
| pYC2/CT | Invitrogen |

1. Weimer PJ, Wagner LW, Knowlton S, Ng TK: **Thermophilic anaerobic bacteria which ferment hemicellulose: characterization of organisms and identification of plasmids.** *Arch Microbiol* 1984, **138:**31-36.

2. Sullivan MA, Yasbin RE, Young FE: **New shuttle vectors for Bacillus subtilis and Escherichia coli which allow rapid detection of inserted fragments.** *Gene* 1984, **29:**21-26.

3. Olson DG, Tripathi SA, Giannone RJ, Lo J, Caiazza NC, Hogsett DA, Hettich RL, Guss AM, Dubrovsky G, Lynd LR: **Deletion of the Cel48S cellulase from Clostridium thermocellum.** *Proc Natl Acad Sci U S A*.

**Supplemental Table 2.** Primers used in plasmid construction.

| Primers | Sequence |
| --- | --- |
| Cthe_pyrF_rev+40bp_pYC2 | TAA TTA CAT GAT GCG GCC CTC TAG GAT CAG CGG GTT TAA ACG CTG AGG CGC GCC GAA TTC GGT ACC AAA ACA AAA GGC CCA GTC TTC CGA CTG AGC CTT TTG TTT TCT CGA GGC CCG GGC TTA CTT CCT GTC TCG CAA CG |
| Cthe_pyrF_for+40bp_cat | TTT ATA ATA AAG GAG GTC GAC GTC ATG TTT ATT GAT ACA TTA ATT GAA AAG ATT AGA GA |
| P-gapD-cat for+40bp_pYC2 | CGC GCT TAA TGG GGC GCT ACA GGG CGC GTG GGG ATG ATC CAC TAG TAA GCT TGG ATC CTC GCG AGG CCG GCC AGT ATT CTG ACA TGG GTG |
| P-gapD-cat rev+40bp_pyrF | TTA ATG TAT CAA TAA ACA TGA CGT CGA CCT CCT TTA TTA TAA AAG CCA GTC ATT AG |
| Tsacch-hpt-for+40bp_cat | ATA GGC CTA ATG ACT GGC TTT TAT AAT AAA GGA GGT CGA CGT CAT GGA AAA TTT ATC AAA AGA CAT CGA TGA AAT TT |
| Tsacch-hpt-rev+40 bp_pAMG202 | CCC AGT CTT CCG ACT GAG CCT TTT GTT TTC TCG AGG CCC GGG CTT AGC TGT ACA TTT CAG GTT TCA AAA CGC C |
| pNW33Nori-for+40bp_pAMG202SpeI | ATG GGG CGC TAC AGG GCG CGT GGG GAT GAT CCA CTA GTG AAT TTA GGA GGC TTA CTT GTC |
| pNW33Nori-rev+40bp_pAMG202SpeI | TGT CAG AAT ACT GGC CGG CCT CGC GAG GAT CCA AGC TTT ATG GGA AAC AAA ATA TTG CGT |
| pyrF internal to del dcm-f^a^ | GAAATTCAGGACATACTCAC**G**CAGGAAGGAAGAAGCATTTATG |
| pyrF internal to del dcm-r^a^ | CATAAATGCTTCTTCCTTCCTG**C**GTGAGTATGTCCTGAATTTC |
| pYC2-CT-seq-r | GGCGTGAATGTAAGCGTGACATAA |
| cat-seq-out-f | GGTTATCATGCAGGATTGTTTATGAACT |

^a^ Bold, underlined base is the base that is mutated to eliminate Dcm methylation but maintain the amino acid sequence.
